# Deconstructing post-exertional malaise in myalgic encephalomyelitis/ chronic fatigue syndrome: A patient-centered, cross-sectional survey

**DOI:** 10.1371/journal.pone.0197811

**Published:** 2018-06-01

**Authors:** Lily Chu, Ian J. Valencia, Donn W. Garvert, Jose G. Montoya

**Affiliations:** Stanford ME/CFS Initiative, Division of Infectious Diseases and Geographic Medicine, Stanford University School of Medicine, Stanford, California, United States of America; Universitat Wien, AUSTRIA

## Abstract

**Background:**

Post-exertional malaise (PEM) is considered to be the hallmark characteristic of myalgic encephalomyelitis/ chronic fatigue syndrome (ME/CFS). Yet, patients have rarely been asked in formal studies to describe their experience of PEM.

**Objectives:**

To describe symptoms associated with and the time course of PEM

**Methods:**

One hundred and fifty subjects, diagnosed via the 1994 Fukuda CFS criteria, completed a survey concerning 11 symptoms they could experience after exposure to two different types of triggers. We also inquired about onset and duration of PEM and included space for subjects to write in any additional symptoms. Results were summarized with descriptive statistics; McNemar’s, paired t-, Fisher’s exact and chi-square goodness-of-fit tests were used to assess for statistical significance.

**Results:**

One hundred and twenty-nine subjects (90%) experienced PEM with both physical and cognitive exertion and emotional distress. Almost all were affected by exertion but 14 (10%) reported no effect with emotion. Fatigue was the most commonly exacerbated symptom but cognitive difficulties, sleep disturbances, headaches, muscle pain, and flu-like feelings were cited by over 30% of subjects. Sixty percent of subjects experienced at least one inflammatory/ immune-related symptom. Subjects also cited gastrointestinal, orthostatic, mood-related, neurologic and other symptoms. Exertion precipitated significantly more symptoms than emotional distress (7±2.8 vs. 5±3.3 symptoms (median, standard deviation), p<0.001). Onset and duration of PEM varied for most subjects. However, 11% reported a consistent post-trigger delay of at least 24 hours before onset and 84% endure PEM for 24 hours or more.

**Conclusions:**

This study provides exact symptom and time patterns for PEM that is generated in the course of patients’ lives. PEM involves exacerbation of multiple, atypical symptoms, is occasionally delayed, and persists for extended periods. Highlighting these characteristics may improve diagnosis of ME/CFS. Incorporating them into the design of future research will accelerate our understanding of ME/CFS.

## Introduction

Myalgic encephalomyelitis/ chronic fatigue syndrome (ME/CFS) is a common, chronic, complex, medical condition that affects at least one million people in the United States, equal to or more than multiple sclerosis, rheumatoid arthritis, or systemic lupus erythematosus [1]. Despite its high prevalence and disabling nature, up to 91% of those affected [2] are estimated to be undiagnosed or misdiagnosed and the remainder of patients often report months and years of visiting multiple doctors [3] before arriving at an answer. Forty-eight percent of clinicians do not feel confident about making a diagnosis of ME/CFS, 67% believe it is more difficult to diagnose than other conditions and nearly this same percentage holds even among clinicians who have made the diagnosis at least once before [4–6].

### Origin and evolution of PEM

One reason for this lack of certitude may be traced back to how ME/CFS is defined. The most commonly used ME/CFS definition for research and clinical care is the Fukuda chronic fatigue syndrome (CFS) criteria [7], created by the United States’ (US) Centers for Disease Control and Prevention (CDC) in 1994. To be diagnosed, patients must not only suffer from unrelenting or recurrent, function-limiting fatigue unresponsive to rest for 6 months, they must also have experienced at least four out of the following eight symptoms concurrently: unrefreshing sleep, tender cervical/ axillary lymph nodes, muscle pain, multi-joint pain without signs of inflammation, sore throat, impaired memory or concentration, new or changed headaches, and post-exertional malaise (PEM). Many of these same symptoms occur in healthy people transiently and in multiple medical conditions (e.g. major depression, fibromyalgia, sleep apnea, various viral infections). Consequently, many clinicians, concerned about missing an alternative diagnosis, are reluctant to diagnose someone with ME/CFS.

The one Fukuda symptom that is unique is post-exertional malaise: this term was coined by Fukuda et al. and so had no prior medical meaning attached to it. Yet, for over two decades, the CDC did not define PEM at all, inadvertently opening up the term to potentially inaccurate and inconsistent interpretations by clinicians, researchers, and patients. In 2001, King et. al. [8] wrote “no clear operational definition of this symptom exists” and that, for example, “some clinicians may require worsening of…three…symptoms…while others may just require a worsening of fatigue” after exertion. A 2003 article [9] co-authored by CDC staff purporting to identify ambiguities in the Fukuda criteria did not discuss PEM but rather focused on fatigue definitions and scales. Adding to the confusion, the word “malaise” itself is vague. The Oxford English Dictionary [10] defines “malaise” as “a general feeling of discomfort, illness, or unease whose exact cause is difficult to identify.”

While their website is gradually being revised [11], for the last 5 years, CDC has described PEM as “increased malaise (extreme exhaustion and sickness) following physical activity or mental exertion” [12]. The word “sickness” is broad and ambiguous. Thus, it is not surprising that clinicians have had difficulty understanding PEM and using the symptom to help distinguish ME/CFS from other medical conditions. In the broadest sense, PEM could refer to any symptom that occurs after exertion, whether it is muscle pain related to short-term overexertion in a healthy person, shortness of breath due to asthma, or chest pain due to heart disease. Researchers have been similarly confused, conflating PEM with post-exertional fatigue [13, 14] or avoiding it altogether. Counting even superficial evaluations, only a handful of clinical trials (for example, [15, 16]) have included post-exertional malaise as an outcome. Lastly, if the term PEM is used but not explained to subjects in studies, there is no way to tell if what they understand PEM to be corresponds to what researchers or clinicians conceive it to be. Jason et al. [17] have shown that the numbers of subjects affirming they have PEM can vary from as little as 41% to as high as 94% depending on how they are asked. For example, they found that some subjects confirming PEM on further questioning would initially replied “No” if they were questioned about PEM “after…exercise.” These subjects experienced PEM from merely carrying out activities of daily living (e.g. dressing, cooking, etc.) and thus did not exercise anymore.

### Alternative perspectives

In contrast to the CDC, many researchers and clinicians specializing in ME/CFS, as well as patients, have long championed post-exertional symptom exacerbation to not just be one but the hallmark characteristic of ME/CFS. As early as the 1950s, Dr. Melvin Ramsey, the infectious disease physician who created the term “myalgic encephalomyelitis” after seeing multiple patients with the same unusual presentation, considered “as the sheet anchor of diagnosis”, “muscle fatigability, whereby, even after a minor degree of physical effort, three, four or five days, or longer, elapse before full muscle power is restored” [18]. These parties believe the Fukuda definition’s emphasis on the ubiquitous symptom of fatigue as the principal and only mandatory symptom (none of the other eight auxiliary symptoms are required as long as any four symptoms are present) to be fallacious, resulting in both lack of and erroneous diagnoses by clinicians and inclusion of patients into studies who were not truly affected by ME/CFS. Subsequently, they developed three other clinical case definitions: the Canadian Consensus Criteria (CCC, 2003 and 2010 versions), the Myalgic Encephalomyelitis–International Consensus Criteria (ME-ICC, 2011), and Systemic Exertion Intolerance Disease (SEID, 2015) [19–22].

Although these three definitions vary, they all require some version of post-exertional symptom exacerbation, with the ME-ICC calling this phenomenon “post-exertional neuroimmune exhaustion” or PENE and the CCC and SEID retaining the term PEM. Whatever the term, each definition identifies both cognitive and physical exertion as impetuses of PEM, provides a list of specific symptoms beyond post-exertional fatigue (e.g. flu-like feelings, pain, sore throat, cognitive problems, decreased functional capacity), indicates that PEM could vary depending on the individual, and remarks on the sometimes delayed and prolonged nature of PEM. Two important differences exist between the Fukuda and the other three criteria. The former was originally formulated for research purposes and did not evolve much with time or scientific studies whereas the latter three were developed for clinical diagnosis and, therefore, also incorporated the accumulated experiences of patients and clinicians taking care of thousands of people. For the purposes of this paper, we will use “PEM” henceforth to denote PEM, PENE, or concepts related to post-exertional symptom exacerbation.

### Past research: Gaps and opportunities

Nevertheless, despite the focus on PEM by many people in the ME/CFS community, hardly any studies have asked patients directly, open-endedly, and comprehensively about their experience of PEM. In 2012, Haywood and colleagues [23] reviewed 77 different patient-reported outcome measures used previously in ME/CFS studies and concluded, except for the SF-36, all measures were limited in their evidence base. Although PEM was not especially mentioned, they emphasized that “discrepancies exist between what is measured in research and how patients define their experience of CFS/ME” and that investigators “must seek to involve patients more collaboratively.” This predicament is echoed by the US Food and Drug Administration’s 2014 document, Guidance for Industry ME/CFS: Developing Drug Products for Treatment [24], meant to encourage and guide pharmaceutical innovation. The FDA concluded that there were no “patient-report instruments optimal for measurement of fatigue or other symptoms” of ME/CFS.

To date, researchers have questioned patients primarily about post-exertional fatigue [25, 26] and sometimes pain [27, 28] but rarely, about other post-exertional symptoms [29, 30], yet those symptoms may be equally or more disabling for patients than pain or fatigue [31]. Symptom-associated questionnaires, instruments, or tests (e.g. polysomnography, actigraphy, neuropsychological tests) are chosen by researchers based on their own preconceptions about PEM and, usually, only one or two symptoms are addressed in each study. Moreover, researchers have assessed PEM-related outcomes on a fixed schedule (e.g. 1 hour, 24 hours, etc.) after a challenging stimulus [32, 33], rather than matching the time of measurement to the PEM course reported by individual subjects. Most studies also follow subjects for less than 3 days after a PEM-eliciting challenge despite evidence that PEM can endure much longer [34]. Inattention to timing may lead to potential omission or imprecise characterization of important findings at the beginning, peak, or end of PEM. While these arrangements might have been instituted for logistical, practical, financial, or other reasons, they may have impeded a through exploration of PEM and led to inaccurate assumptions about the nature of PEM, especially among physicians and researchers outside the ME/CFS field.

An exception to the situation are two small case-control studies [35, 36] from the Workwell Foundation. After being subjected to 2 cardiopulmonary exercise tests (CPET) separated by 24 hours, a total of 41 female ME/CFS subjects completed an open-ended questionnaire about their symptoms for up to a week after the tests. Thirty-seven healthy, sedentary subjects served as controls. The researchers examined their responses and coded them under symptom categories, as dictated by the CCC. The ME/CFS subjects experienced some symptoms the healthy subjects did not experience at all (e.g. lightheadedness, sore throat/ swollen glands, cognitive dysfunction) and were significantly more likely to experience other symptoms (e.g. pain (odds ratio (OR) = 15.7, p<0.01) and sleep disturbance (OR = 37.5, p<0.001)) compared to their healthy counterparts. Using a binary logistic regression model, only 4 post-exertional symptoms (fatigue-, immune-, sleep-, and pain-associated symptoms) were needed to classify 92% of ME/CFS and 88% of healthy subjects accurately. The time course of symptoms also differed substantially: most control subjects experienced the peak of their symptoms on the day of the test and 87%-95% of them had recovered fully by 24 hours after the tests whereas some ME/CFS subjects’ symptoms peaked 24 or 48 hours after the test with 45%-60% of ME/CFS subjects still feeling the effects after 5 days. Workwell found that if a subject were unable to recover fully within 24 hours, the positive likelihood ratio of them being classified as having ME/CFS was 11.4 whereas the negative likelihood ratio was 0.22. If a subject has this characteristic, it increases the clinician’s baseline estimate of disease presence by about 45% whereas if they do not have this trait, their chances decrease by about 30%.

These two studies suggest that the presence of certain post-exertional symptoms and their time course may help diagnose ME/CFS but these findings need replication. Historically, many promising initial results from ME/CFS studies have been contradicted or attenuated by subsequent studies [22]. Consistent results from multiple, independent research groups in different settings can strengthen the validity of findings. Workwell’s studies consisted of younger women (average age 31.5 ± 9.0 years [35]) who were able and willing to complete 2 maximal exercise tests scheduled within 24 hours. Yet, about one-third of ME/CFS patients are estimated to be male and the illness is most common between the ages of 40 and 60 [37]. Furthermore, a significant portion of patients are unable to withstand even a single maximal exercise test [22] and even relatively mild activity like dressing or driving have been reported to cause PEM in patient accounts [38]. Would a more representative group of patients engaging in less demanding tasks contend with a similar pattern of symptoms? Finally, while an open-ended questionnaire is indispensable when beginning to document a phenomenon, such a format may require more time and effort from patients to complete and clinicians and researcher to analyze when used in large studies or in a busy clinic. Open-ended answers may also be interpreted in a variable manner. We wondered whether a close-ended questionnaire supplemented by opportunities to write in narrative answers would produce comparable responses.

Consequently, the main purpose of our study is to describe the symptoms and time course of PEM in a systematic manner by surveying a large number of subjects afflicted by ME/CFS with both close- and open-ended questions. We hypothesized our results would support prior descriptions of PEM and the Workwell Foundation’s findings but provide additional details regarding specific symptoms and timing. We also asked subjects about their reactions to physical and cognitive effort versus emotional distress to determine if there are any differences in presentation of PEM when different stressors are applied. We were able to accomplish these objectives.

## Methods

### Subject recruitment

From March 2010 to August 2011, we recruited 200 subjects affected by ME/CFS and residing in the Northern California as part of our GEISD (Genetic Expression and Immune System Dynamics) study. The purpose of this study was to explore how infectious, immune, and genetic factors interacted in ME/CFS. Subjects consisted of patients from our ME/CFS clinic, people waiting to be seen at the clinic, members of online ME/CFS forums, and participants of local ME/CFS support groups. All subjects were screened using a standardized telephone interview and included if they fitted Fukuda 1994 CFS criteria, were at least 14 years old, were non-pregnant, and had not been exposed to more than 2 weeks of antibiotics or antivirals recently. Subjects were excluded if they were affected by other medical or psychiatric conditions that could explain their ME/CFS symptoms, suffered from certain immunological conditions, struggled with substance abuse issues in the last year (not including nicotine/ caffeine), had received an influenza vaccination within the past 4 weeks, or were unable to communicate in English. Potential subjects were also asked to report whether their ME/CFS onset was related to a viral infection, to rate their physical and cognitive function now compared to before they became sick (on a scale of 0%-100%, with 100% denoting pre-illness function), and to complete two common instruments used to measure fatigue, the Multidimensional Fatigue Inventory (MFI-20, with a summary score ranging from 0–100 with a higher score denoting increased fatigue) [39] and the Fatigue Severity Scale (FSS, with 9 items scored from 0–7, with a higher average score meaning increased fatigue) [40].

### Survey

In 2012, we used the Research Electronic Data Capture (REDCap) web application (http://project-redcap.org/) to design an online survey asking subjects about the history and course of their ME/CFS. We created an initial version of the questionnaire based on our clinical experiences and knowledge of the literature. Next, a focus group of 5 patients reviewed the draft and gave feedback on relevance, content, and format. After reviewing their comments, we composed a final version of the survey. This study was reviewed and approved by the Stanford University Institutional Review Board (Review Panel 3, Protocol 24244). The aforementioned 200 subjects were re-contacted from January 2013 to July 2013 and asked if they wished to participate in our survey. Informed consent was obtained in writing and subjects were given an individualized secured hyperlink to access the survey. A paper version of the survey was also offered in case of technical or cognitive difficulties. After completion, subjects submitted the survey electronically or mailed the survey back to staff. Study staff then manually entered responses from the paper version into REDCap.

To investigate PEM, we asked subjects 4 questions: 1) What symptoms, if any, are triggered or worsened by physical or mental activity?; 2) What symptoms are triggered or worsened by emotional distress?; 3) How soon usually after starting mental or physical exertion does your illness begin to worsen?; and 4) If you feel worse after activities, how long does this worsening usually last? (S1 File) We intentionally refrained from using the word “post-exertional malaise” to try to decrease the chances that subjects already diagnosed with ME/CFS would automatically answer our question based on their knowledge of or preconceived notions about PEM. We also wanted to avoid the term “malaise” and focus on symptoms that occurred or were exacerbated after a specific stressor, not baseline symptoms. For items 1 and 2, subjects were directed to indicate as many of 11 listed symptoms as were exacerbated or to check “none of the above.” We also included an option “Other” with an accompanying text box for them to type/ write in symptoms we had not listed. For items 3 and 4, subjects chose one answer from a choice of time periods (e.g. “Immediately”, “A few hours”, etc.), “It can vary”, or “Not applicable.” If they chose “It can vary,” they were asked to fill in a text box with a range of times.

### Statistical analysis

Data were extracted from REDCap and exported into Microsoft Excel 2013. To assess whether and how survey responders differed significantly from survey non-responders, we compared these two groups based on characteristics collected during the recruitment process. T-tests were used to compare continuous data whereas the chi-square or Freeman-Halton extension of Fisher’s exact test [41] were used for categorical data due to some cells having low counts.

Initially, the number and percentage of subjects choosing various answers to the two different stressors and the total number of symptoms chosen by subjects were calculated. We also reviewed any open-ended answers submitted by subjects and classified them into symptom categories. Next, we created two groups of symptoms and calculated what number and percentage of subjects would fit into those two groups or variations of those groups. To qualify for the first group, labelled “immune/ inflammatory-related”, an individual subject had to have flu-like feelings, a sore throat, and/or tender lymph nodes. For the second group, named “4-symptom PEM” and based on the findings of the Workwell Foundation [31], an individual subject was required to have fatigue, at least one “immune-related” symptom (i.e. flu-like feelings, sore throat, tender lymph nodes), at least one pain-related symptom (i.e. muscle pain, joint aches, headache, sore throat, tender lymph nodes), and sleep disturbance. To evaluate how the number and type of PEM symptoms might differ depending on the trigger, we used a paired t-test and McNemar’s mid-p tests [42], due to the small sample size in some analyses.

We used descriptive statistics to characterize the onset and duration of PEM. To test whether responses about time were statistically significant, we used a one-sample chi-square goodness-of-fit test with the null hypothesis that if onset and duration times were random, the frequency of answers would be distributed evenly across categories. For subjects who chose the answer ‘It can vary” to these items, we examined their written-in time ranges and classified them into specific categories.

Because multiple comparisons were made in this study, we designated a Bonferroni-corrected two-tailed p-value of equal or lesser than 0.003 as significant.

## Results

One hundred and fifty subjects (75% of 200 contacted) subjects responded to the survey. Based on items collected during the recruitment process (S1 Table), survey respondents were more likely than non-respondents to endorse tender lymph nodes (67% vs. 48%, p = 0.06) or post-exertional malaise (99% vs. 92%, p = 0.02). However, this result needs to be interpreted with caution as many subjects did not know what enlarged lymph nodes were and thus might have marked “Unknown” even as they experienced this symptom. There were no significant differences in percentage of women, mean age, duration of illness, prevalence of self-identified viral onset, fatigue scores (using the Fatigue Severity Scale and Multidimensional Fatigue Inventory-20), self-assessed cognitive/ physical functioning, and prevalence of other Fukuda criteria symptoms between responders and non-responders.

One hundred and forty-four subjects completed both items concerning symptoms (items 1 and 2) while 145 and 146 subjects, respectively, completed items about PEM onset and duration (items 3 and 4). Since the percentage of missing responses for each item is low, ranging from 3%-6%, this study simply focuses on the replies that were received. Although the lower age limit of our recruitment criteria was 14, no subject under 18 years of age was included in the final study sample. Men constituted 20% of our subjects and the mean age was 51.6 ± 12.5 years.

Table 1 illustrates the percentage of subjects who reported various symptoms after exposure to two types of stimuli. Most subjects (N = 129, 90%) experienced PEM with both physical/ cognitive exertion and emotional distress. Almost all (N = 143, 99%) were affected by the former trigger but 14 (10%) reported no effect with the latter trigger. Exertion precipitated significantly more symptoms than emotional distress (7±2.8 vs. 5±3.3 symptoms (median, standard deviation), p<0.001). In both situations, fatigue was the most commonly exacerbated symptom, reported by 94% (N = 135) and 76% (N = 109) of subjects, but cognitive difficulties, sleep disturbances, headaches, muscle pain, and flu-like feelings were cited by over 30% of subjects. Sixty (N = 87) and thirty-six percent (N = 52) of subjects experienced at least one inflammatory/ immune-related symptom (flu-like feelings, sore throat and tender lymph nodes) respectively, with either exertion or emotional distress. Approximately a quarter of subjects (N = 33, 23%) recounted aggravation of fatigue and all three inflammatory/ immune-related symptoms after physical or cognitive effort.

**Table 1:** Post-exertional malaise (PEM) symptoms precipitated by physical/ cognitive exertion versus emotional distress.

Symptom	Physical/ cognitive exertion N = 144 (%)	Emotional distress N = 144 (%)	Percent difference in subjects endorsing symptom	p-value^d^
**None**	1 (1%)	14 (10%)	9%	<0.001
**Fatigue**	135 (94%)	109 (76%)	18%	<0.001
**Poor concentration**	112 (78%)	94 (65%)	13%	0.001
**Difficulty thinking**	106 (74%)	88 (61%)	13%	0.002
**Muscle pain**	106 (74%)	48 (33%)	41%	<0.001
**Sleep disturbance**	97 (67%)	95 (66%)	1%	0.77
**Poor memory**	98 (68%)	75 (52%)	16%	<0.001
**Flu-like feelings**	88 (61%)	47 (33%)	28%	<0.001
**Joint pain**	77 (53%)	30 (21%)	32%	<0.001
**Headache**	73 (51%)	53 (37%)	14%	<0.001
**Sore Throat**	60 (42%)	28 (19%)	23%	<0.001
**Tender lymph nodes**	58 (40%)	21 (15%)	25%	<0.001
**Other**^**a**^	29 (20%)	20 (14%)	6%	0.03
**At least 1 immune-/ inflammatory related symptoms**^**b**^	87 (60%)	52 (36%)	24%	<0.001
**Fatigue and all 3 immune-/ inflammatory related symptoms**	33 (23%)	14 (10%)	13%	<0.001
**4-symptom PEM**^**c**^	60 (42%)	30 (21%)	21%	<0.001
**Number of symptoms (median, SD)**	7 ± 2.8	5 ±3.3		<0.001

SD = standard deviation

^a^ Symptoms written in by subjects included: gastrointestinal symptoms, dizziness, pre-syncopal feelings, tingling skin, muscle twitches, sensory overload, anxiety, depression, and feelings of “inflammation.”

^b^ Flu-like feelings, sore throat, tender lymph nodes.

^c^ Per VanNess et al. [35], consists of fatigue, sleep disturbance, at least one pain symptom, and at least one immune-related symptom.

^d^ Except for sleep disturbance and “Other” symptoms, the percentage of subjects experiencing any symptom or group of symptoms after physical/ cognitive exertion is always significantly higher than when the same subjects were exposed to emotional distress. For this study, a p-value of <0.003 was deemed significant.

Outside of the 11 symptoms listed, some subjects also cited gut- (e.g. nausea, irritable bowel; N = 6 and 5, respectively, with exertion vs. emotional distress), orthostatic- (e.g. dizziness, pre-syncopal feelings; N = 4, 3), mood- (e.g. anxiety, depression; N = 4, 4), nervous system- (e.g. sensory overload, tingling skin; N = 12, 7), pain- (e.g. sinus pressure; N = 7, 5), respiratory- (N = 2, 2) and “infection”-related (N = 3, 2) symptoms as components of their PEM. Except for sleep disturbance and “Other” symptoms, exertional stressors were significantly more likely than emotional distress to provoke the enumerated symptoms in subjects (p<0.003). The largest differences in symptoms provoked were related to musculoskeletal pain (41% difference for muscle pain and 32% difference for joint pain) and immune/ inflammatory-related symptoms (23% to 28% difference). When we applied the Workwell Foundation’s “4-symptom PEM” schema, a significantly higher percentage of subjects experienced this set of symptoms with exertion while only 21% did with emotional distress (42% vs 21%, p<0.001).

**Fig 1:**
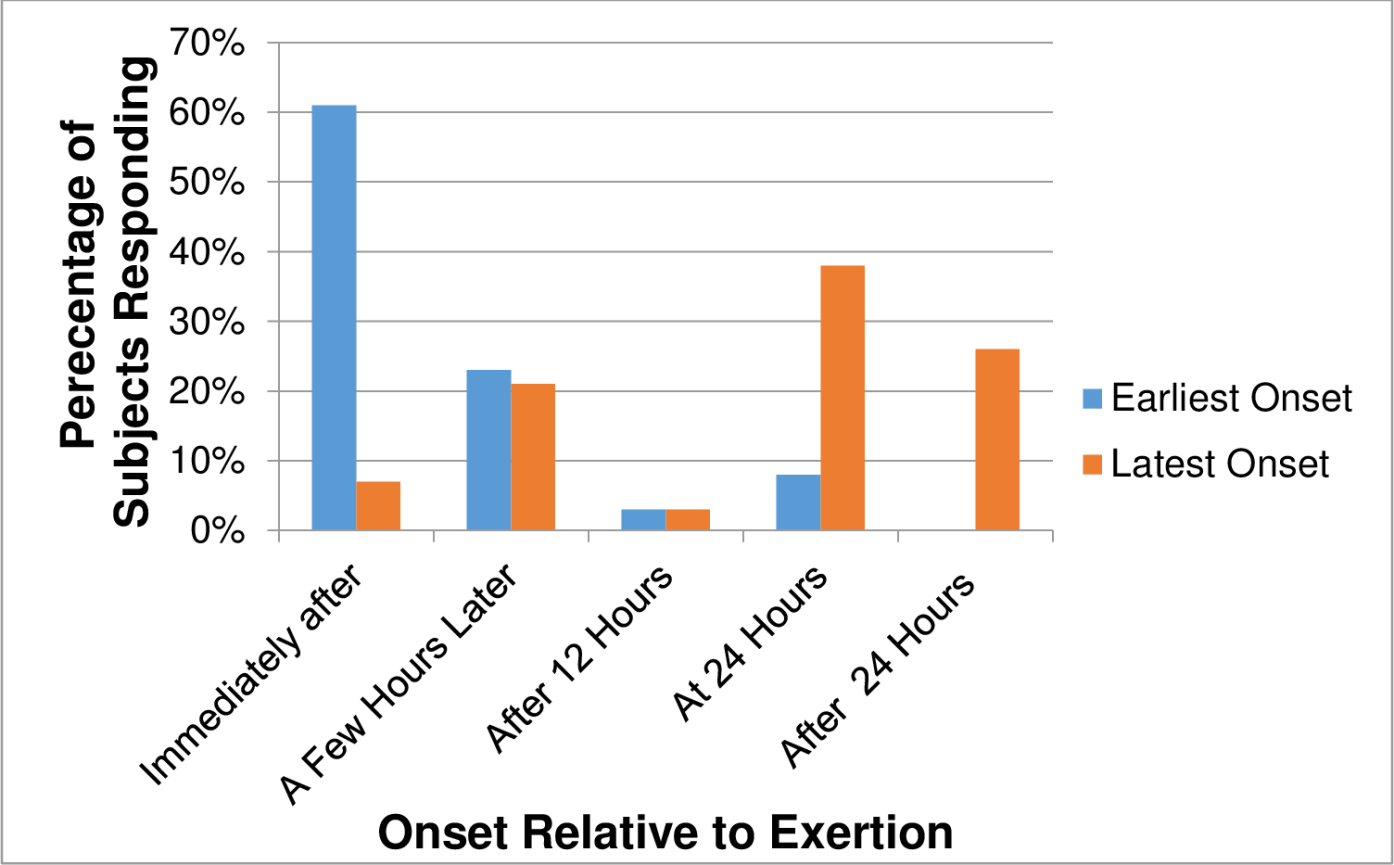
Range of PEM onset times after physical/ cognitive exertion for subjects (N = 61) with inconsistent times^a^. ^a^ These subjects chose the answer “It can vary” when queried about when their PEM began relative to an exertional trigger. Space was provided for them to write down the earliest and latest times their PEM could start. Three subjects noted their onset times fluctuated so frequently they were unable to even give a time range.

**Fig 2:**
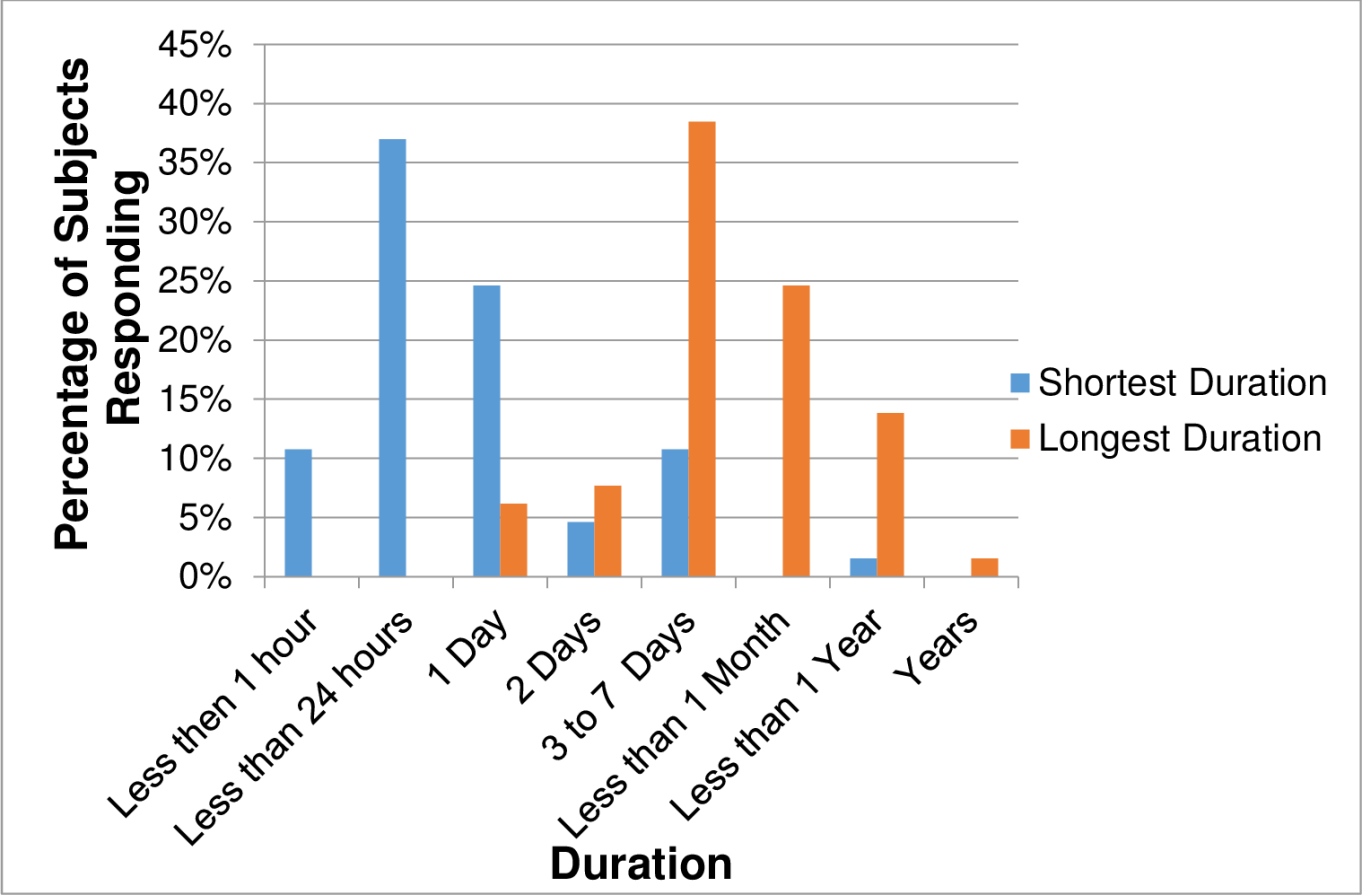
Range of duration of PEM after physical/ cognitive activities for subjects (N = 65) endorsing inconsistent times^a^. ^a^ These subjects chose the answer “It can vary” when queried about how long their PEM lasted after physical or cognitive activities. Space was provided for them to write down the shortest and longest times their PEM could be sustained. Five subjects did not give a specific time range.

Table 2 presents information regarding the onset and duration of PEM after physical or cognitive exertion. The most common answer, given by approximately 42% (N = 61, out of a total of 145) and 45% (N = 65, out of a total of 146) of subjects respectively, was that initiation and duration of PEM varied. For these groups, the most common earliest time of onset was immediately (N = 37, out of 61, 61%) and the most common latest time of onset was 24 hours later (N = 23; 38%) (Fig 1); the most common shortest PEM duration was a few hours (N = 23, out of 65, 35%) with the most common longest PEM duration being three to seven days (N = 25, 38%) (Fig 2). A substantial number of subjects imparted that their PEM could last as long as weeks (N = 16, 25%) or even months (N = 9, 14%). A few commented that their time patterns varied so much it was difficult even to put down a range; variations frequently depended on the type and intensity of activity.

**Table 2:** Onset and duration of post-exertional malaise (PEM) after physical/ cognitive exertion.

Length of time to PEM onset post-exertion^a^ (N = 145)	N (%)	Duration of PEM post-exertion^b^ (N = 146)	N (%)
**Immediately**	23 (16%)	**1–6 hours**	2 (1%)
**About 1 hour later**	13 (9%)	**6–12 hours**	3 (2%)
**1–3 hours later**	12 (8%)	**12–24 hours**	9 (6%)
**3–24 hours later**	10 (7%)	**1 day**	11 (8%)
**More than 24 hours later**	16 (11%)	**2 days**	18 (12%)
**It can vary**	61 (42%)	**3–7 days**	29 (20%)
**Not sure**	7 (5%)	**More than 1 week**	5 (3%)
**Not applicable**	3 (2%)	**It can vary**	65 (45%)
		**Not applicable**	4 (3%)

^a^ p<0.001 for onset with chi-square goodness-of-fit one-sample test if null hypothesis is equal proportions for each category

^b^ p<0.001 for duration with chi-square goodness-of-fit one-sample test if null hypothesis is equal proportions for each category

Of those subjects choosing the other time categories, 40% of subjects reported PEM consistently beginning within 24 hours of exertion but 11% reported a consistent post-trigger delay of at least 24 hours. PEM abated within 24 hours routinely in only 9% (N = 14) of subjects. Another 20% of subjects endured PEM for 1 to 2 days but a quarter noted that PEM persisted for more than 3 days. A small percentage of subjects, 2% (N = 3) and 3% (N = 4), respectively, selected the choice “Not Applicable” when asked about onset and time; this is surprising since for all but one subject, the same individual, in each category, had confirmed symptom trigger or exacerbation with exertion. A one-sample chi-square goodness-of-fit test returned a p-value of p<0.001, suggesting that the distribution of onset and duration answers was unlikely to occur simply based on chance.

## Discussion

This the first study to directly and systematically ask a large sample of ME/CFS subjects about how they experience PEM in the course of their lives. We found that a more representative sample of study participants, including men and older patients, suffered from a similar cluster and timeline of PEM symptoms under usual life circumstances as younger women subjected experimentally to two bouts of maximal exercise [35,36]. Since most daily activities do not involve maximal levels of exertion, our results provide formal evidence supporting patient narratives [38], clinician experiences, and current case definitions [19–22] which assert that even tasks like walking, cooking, or reading can provoke PEM. Second, we documented for the first time that emotional distress can provoke a variety of symptoms and that symptom prevalence may differ depending on the trigger. Third, we reinforced that timing of PEM can vary both between and within individuals and supplied specific ranges of times. Finally, we demonstrated that a simple, close-ended questionnaire may be feasible for capturing the principle features of PEM.

The overwhelming majority (90%) of subjects experienced PEM with physical/ cognitive exertion and emotional upheaval. Aggravation of multiple symptoms, a median of 7 and 5, respectively, was the rule rather than the exception. For 8 out of 11 symptoms, at least a third of subjects encountered them under either situation (Table 1). Some symptoms are commonly observed with physical exertion even in healthy people (e.g. muscle pain) while others are atypical (e.g. flu-like feelings, gut-related symptoms, sensory overload), neither reported by healthy people nor people affected by other medical conditions. Because our questionnaire listed specific symptoms rather than combinations (e.g. “sore throat/ tender lymph nodes” [35]) or categories of symptoms (e.g. “autonomic” [36]), a direct comparison to Workwell’s findings is not possible. Nevertheless, the multitude of symptoms recollected by our subjects reflect those of Workwell’s subjects. For example, fatigue-, pain-, and sleep-related symptoms were the top 3 categories noted by Davenport [36], analogous to the first, fourth, and fifth most common symptoms observed by us post-exertion (fatigue, muscle pain, sleep disturbances). Other symptom combinations they used such as “light-headedness/ vertigo” were not a part of the 11 symptoms we listed but emerged in the open-ended portion of our questionnaire.

Contrary to some sources which have intimated that patients affected by ME/CFS are reluctant to admit the role of psychological or emotional factors in their illness and cling unreasonably to a biological cause for their condition [43–45], our clinical experience, supported by this study’s results, is that patients readily discuss such factors when their illness experiences are validated. Subjects experienced the same array of diverse symptoms with emotional distress as provoked by exertion albeit at a lower rate. They also encountered symptoms such as musculoskeletal pain and sore throat that are usually not linked to emotional distress in healthy people or many people affected by other conditions.

PEM onset and duration varied both between and within individuals. Table 2 shows a fairly even distribution of answers across individuals except for the most dominant answer, “It can vary” selected by a little over 40% of study participants. Although it may appear that only a minority of subjects reported PEM onset 24 hours after a trigger, the number of subjects suffering delayed-onset PEM is higher. Some of these subjects endorsed the “It can vary” category instead if not every episode of their PEM was delayed by 24 hours. Combining the subjects who consistently reported PEM onset after 24 hours (N = 16) with those who reported that their PEM could be delayed 24 hours or more (N = 38, Fig 1) means that potentially, in up to 37% of subjects (N = 54, out of 145), PEM may not begin until a day or more after an exertional trigger. This figure agrees with Van Ness et al.’s paper. Although their paper did not elaborate on delayed onset, their Fig 1 shows that for some subjects, some symptoms did not start until the day following the exercise test. From the limited details in their text, we were able to calculate that muscle pain did not begin for 28% of their subjects until 24 hours had passed [35].

Likewise, the most common answer chosen by respondents when queried about duration was “It can vary.” At least 43% of subjects noted that their PEM regularly lasted more than 24 hours but, as with PEM initiation, this percentage may be higher given that some subjects selected “It can vary” when some PEM bouts are shorter. When those who consistently reported PEM duration of equal or more than 24 hours (N = 63) are combined with those whose PEM occasionally lasts 24 hours or more (N = 60, Fig 2), 123 subjects, or up to 84%, may sustain PEM for a day or more. Only 9% of subjects consistently reported PEM resolving within 24 hours. PEM could last for quite extensive periods. Again, combining consistent with occasional reports, when asked about their longest episodes of PEM, 24% (N = 35) of our sample answered they could last more than a week, up to several months.

It is not surprising that PEM time course fluctuates within individuals. The post-Fukuda case definitions [19–22] as well as patient accounts [22, 38, 46] note that there are variations not only between but within individuals. The threshold needed to trigger, moment of initiation of, severity of, and time span of PEM depends on both the baseline state of individuals (e.g. Did they sleep well the night before? Do they have a concurrent cold? How much total activity did they engage in this week so far?) and the type, intensity, duration, and frequency of the trigger applied. As one patient who is also a physician described it, the “safety zone” for avoiding PEM “moves around” [46] and this unpredictability contributes to patients’ problems scheduling and participating in occupational, educational, recreational, social, and personal care activities.

Overall, our questionnaire performed reasonably well. The number of missing responses was low and we received few questions from study participants about survey items. Our symptom list appears to have covered the most common PEM symptoms as no spontaneously volunteered symptom affected more than 8% of our subjects.

### Clinical implications

Based on our data, we suggest that educational materials targeted at clinicians highlight characteristics that distinguish PEM from the post-exertional or emotionally distressing experiences of healthy people and people with other illnesses. First, most people may cite one or another factor as exacerbating their symptoms but it is unlikely someone without ME/CFS will cite physical exertion, cognitive effort, and psychological stress as causing the same or similar constellation of symptoms. Most patients will bring up a symptom complex suspicious for PEM linked to physical exertion. Clinicians should ask if there are any other triggers and if none are offered, ask patients particularly about the effects of cognitive exertion and emotional situations.

Second, the concurrence and type of symptoms aggravated are a strong clue to PEM. Post-exertional fatigue, muscle pain, and joint aches are very common after even minor activity in many people so mere presence of these three symptoms may not help clinicians recognize PEM. However, other symptoms associated with PEM are either not usually linked to exertion/ emotional distress or even paradoxically improved by physical exertion in persons unaffected by ME/CFS. For example, there exists no medical condition the authors are familiar with where exertion or emotional distress causes immune/ inflammatory-related symptoms like sore throat, tender lymph nodes, or flu-like feelings, yet 60% and 36% of our subjects, respectively, reported these symptoms with either stimuli and about a quarter experienced all 3 with exertion. Conversely, symptoms typically associated with physical exertion in other conditions, like shortness of breath or chest pain in chronic lung or heart disease, are rarely reported in ME/CFS. Furthermore, it is well-established that physical activity improves mood, sleep, and pain in both healthy people as well those with chronic illnesses like depression or anxiety [47–49] yet our subjects report worsened sleep, mood, and pain with physical activity. This paradoxical effect is also demonstrated by studies which focus on the physiological aspects of ME/CFS [25, 50, 51].

Third, PEM has an unusual time course. In many medical conditions, exertion-exacerbated symptoms usually start during exertion or immediately after and usually resolve immediately or shortly after exertion stops. In contrast, PEM may not start until hours or even days after the trigger starts or has been removed, may peak after the first day, and may not stop until hours to months later. This characteristic of PEM often leads patients and clinicians to believe that symptom exacerbations are random rather than associated with a trigger; most people will not intuit that symptoms are caused by a trigger that occurred hours to days prior unless specifically asked by their clinicians to pay attention.

The recent Institute of Medicine report [22] specifically underscored the urgent need to develop simple, practical medical history, questionnaire, or physical examination items that could be used at the bedside to quickly and accurately diagnose ME/CFS. This triad of characteristics–precipitants, number/ type of symptoms, and time course–can be applied now to help clinicians identify PEM, and thus, assist in the diagnosis of ME/CFS. For situations where clinicians are unable to elicit a clear history from a patient, they can ask the patient to keep a short-term diary of trigger and symptom patterns to help clarify matters. Simultaneously, our results could be combined with those of other researchers to produce formal instruments to ascertain for the presence of PEM. Applying the Workwell Foundation’s findings [36], only 42% of our subjects qualified for their “4-symptom PEM” schema. A different set of symptoms, perhaps accompanied by severity ratings, may be needed in order to detect PEM in more patients. In contrast, similar to their figures of 100% and 81% [35, 36], almost all of our subjects (84%) had difficulty recovering within 24 hours. To assess validity and reliability, any instruments generated could be tested against objective measures of PEM such as 2-day repeated cardiopulmonary exercise testing [52–54] or against subjects who have been verified by multiple experienced clinicians as being affected by ME/CFS.

### Research implications

This study also has implications for the design, analysis, and interpretation of future studies. Most studies of PEM have relied on a physical exertion stimulus but our results support patient and clinician reports that other precipitants can lead to PEM. Only a few studies have used cognitive, orthostatic, or psychological triggers [25, 55, 56]. Future studies should continue to explore the effects of other precipitants. Researchers need to expand outcome measures beyond only pain and fatigue and, when screening subjects for PEM, not merely ask if potential subjects have “PEM” but inquire about post-exertional symptoms in more detail. When researchers focus on only one or two symptoms, instead of multiple symptoms, and concentrate on very common symptoms seen even in non-ME/CFS subjects, there is no clear assurance they are studying the phenomenon of PEM as reported by clinicians, patients, the Workwell Foundation, or our study.

Most researchers have logically chosen to evaluate symptoms expected to be elicited by their selected precipitant, e.g. studying physical fatigue after a bout of exercise or problems thinking after a mentally-fatiguing neuropsychological battery. Our survey results indicate it may be worthwhile to consider symptoms outside of those anticipated. For example, scientists could inquire about immune/ inflammatory symptoms and/or measure blood-borne markers of inflammation after subjecting study participants to a cognitively-challenging task like driving [25]. Moreover, researchers should examine the understudied symptoms of PEM like mood-, gut-, immune/ inflammatory-related, or other neurologically-related symptoms like sensory overload. These symptoms are acknowledged in the post-Fukuda case definitions but very little research has been carried out on them. The pathophysiology of PEM would be much advanced by investigating what underlying physiological factors could lead to such a constellation of heterogeneous symptoms.

Finally, researchers need to heed the unusual time course of PEM and its variability among and within patients. Our study participants explained that type, intensity, frequency, and duration of PEM-inducing stimuli can unpredictably influence the expression and timeline of PEM. In some circumstances, PEM may even last for weeks or months. Therefore, studies need to be extended past a few days and, ideally, timing of outcome measures, whether subjective questionnaires or objective testing, should be adjusted to fit individual subjects’ chronology of PEM. Otherwise, researchers may miss crucial moments like the beginning, peak, or end of PEM, where differences between subjects and controls might be unmasked and/or heightened. Delayed initiation of PEM relative to a precipitant is especially peculiar and paying closer attention to this characteristic may yield vital clues to the mechanisms behind PEM. If scientists must measure outcomes at fixed times, they can at least ask subjects to tell them where those times fall within the trajectory of their PEM.

### Limitations

Weaknesses of this study include possible responder bias, a less detailed PEM questionnaire than ideal, and limited generalizability. During the recruitment process, we screened subjects using the Fukuda criteria. Although subjects did not have to have PEM to qualify for the study, 99% of subjects responded affirmatively when asked if they had PEM. Thus, despite our efforts to obtain an unbiased picture of PEM without evoking existing definitions or accounts of PEM, some subjects’ reports of PEM might have been unduly influenced by what they had read or heard previously. In the future, this might be remedied by prospectively interviewing patients at risk for ME/CFS, e.g. soon after an episode of Epstein-Barr infectious mononucleosis, before they have heard of or have become familiar with the term PEM, rather than retrospectively.

Secondly, since this survey was one section of a much longer patient survey, we restricted the number and complexity of survey items. For example, we did not separate physical exertion from cognitive exertion, did not include other types of stressors (e.g. orthostatic), did not inquire about the intensity of stressors, and did not ask specifically when PEM started and when it ended with emotional distress. Jason’s group has suggested that inquiring about the quality or type of post-exertional fatigue [57] and the frequency and intensity of symptoms might help distinguish ME/CFS [58]. For the choices “It can vary”, we could have further standardized this item by asking subjects to choose from a pre-defined range of times rather than having them write in answers and then classifying them post-hoc into categories. Time categories given could be made more distinct, e.g. subjects might be confused by categories such as “1 day” versus “12–24 hours” which overlap. Lastly, the small number of subject answering “Not Applicable” to our time items despite endorsing PEM might be due to the answer choices given, e.g. if a subject’s PEM consistently lasted less than 1 hour, there was no specific choice corresponding to this situation. These are details we will consider for future studies.

Thirdly, since, at most, only 15% of afflicted patients are estimated to have been diagnosed by their attending physician, to assure that an adequate number of subjects were recruited, we drew subjects not only from Stanford University’s ME/CFS specialty clinic but also from the clinic’s waiting list, local support groups, and community announcements. Despite these efforts, our sample population was dominated by self-identified Caucasian (95% of subjects), middle-aged, women. This is also true of most ME/CFS studies [22] and may reflect not just the biology of ME/CFS (studies consistently show women are affected at 2–3 times the rate of men) but also broader socio-economic, gender-related, and even clinician-based trends associated with healthcare access, healthcare seeking, and diagnostic biases. Consequently, our findings may not be as generalizable to groups such as children, men, ethnic minorities, or the poor who are also affected by ME/CFS.

## Conclusion

The major strengths of this study lie in its methodical, patient-centered exploration of PEM in a large sample of subjects affected by ME/CFS. Definitions of PEM have been constructed predominantly from anecdotal reports by clinicians and patients supplemented with valuable but incomplete, inadvertently biased, and/or limited studies. Our study provides exact symptom and time patterns for PEM that is generated in the course of patients’ lives. We supply formal evidence that PEM linked with likely submaximal physical/ cognitive activity shows similar symptoms and time patterns as that preceded by experimentally-administered maximal physical activity. This discovery is important as it supports the daily struggles patients face due to their ME/CFS. Our findings may be used by clinicians to diagnose ME/CFS and by researchers to design more comprehensive studies of PEM. Early, accurate diagnosis of ME/CFS and a thorough understanding of PEM will hopefully accelerate progress toward effective disease-modifying treatments for ME/CFS, none of which exist at the moment.

## Supporting information

S1 File Survey items and responses. (DOCX)

S1 Table Survey non-responder versus responder answers to items assessed during study recruitment. (DOCX)
