# Supplementary material for: Deconstructing post-exertional malaise in myalgic encephalomyelitis/ chronic fatigue syndrome: A patient-centered, cross-sectional survey
Source: PLoS One. 2018 Jun 1;13(6):e0197811. doi: 10.1371/journal.pone.0197811 (PMC5983853; doi:10.1371/journal.pone.0197811)
Supplement: S1 File — (DOCX) [file pone.0197811.s001.docx]

**Survey Items and Responses**

Each item was followed by a choice of responses. For items #1 and #2, study participants could choose as many responses as applied to their individual situation. For items #3 and #4, they were instructed to choose only one answer.

For items #1 and #2, choosing the response “Other” triggered a text box to appear prompting participants to describe what other symptoms they experienced. “If 'other,' please describe:.”

1) What symptoms, if any, are triggered or worsened by physical or mental activity?

a. Fatigue

b. Sore throat

c. Sleep disturbance

d. Headache

e. Poor concentration

f. Poor memory

g. Difficulty thinking

h. Muscle pain

i. Joint pain affecting multiple joints

j. Tender lymph nodes

k. Flu-like feelings

l. None of the above

m. Other

2) What symptoms are triggered or worsened by emotional distress?

a. Fatigue

b. Sore throat

c. Sleep disturbance

d. Headache

e. Poor concentration

f. Poor memory

g. Difficulty thinking

h. Muscle pain

i. Joint pain affecting multiple joints

j. Tender lymph nodes

k. Flu-like feelings

l. None of the above

m. Other

For items #3 and #4, choosing the response “It can vary” triggered a text box to appear prompting study participants to give us a range of times. “If it varies, please give us a range: for example, from three to five hours.”

3) How soon usually after starting mental or physical exertion does your illness begin to worsen?

a. Immediately

b. About 1 hour later

c. From one to three hours later

d. More than three hours later

e. More than 24 hours later

f. It can vary

g. Not sure

h. Not applicable

4) If you feel worse after activities, how long does this worsening usually last?

a. 1 - 6 hours

b. 6 – 12 hours

c. 12 – 24 hours

d. 1 day

e. 2 days

f. 3 – 7 days

g. More than a week

h. It can vary

i. Not applicable
