# Supplementary material for: Deconstructing post-exertional malaise in myalgic encephalomyelitis/ chronic fatigue syndrome: A patient-centered, cross-sectional survey
Source: PLoS One. 2018 Jun 1;13(6):e0197811. doi: 10.1371/journal.pone.0197811 (PMC5983853; doi:10.1371/journal.pone.0197811)
Supplement: S1 Table — (DOCX) [file pone.0197811.s002.docx]

S1 Table. Survey non-responder versus responder answers to items assessed during study recruitment.

|  | **Responded to Questionnaire** | | **p-value^a^** |
| --- | --- | --- | --- |
|  | **No** | **Yes** |  |
| **Total** | 50 | 146^b^ |  |
| **Sex N (%)** |  |  | 0.06 |
| **Female** | 33 (66.0) | 117 (80.1) |  |
| **Male** | 17 (34.0) | 29 (19.9) |  |
| **Age, mean (SD)** | 50.7 (12.9) | 51.6 (12.5)  1 missing | 0.66 |
| **MFI-20, mean (SD)** | 77.8 (12.3) | 73.9 (13.7)  19 missing | 0.08 |
| **FSS Average, mean (SD)** | 6.2 (0.9) | 6.0 (1.1)  17 missing | 0.25 |
| **Duration of Illness, years,**  **mean (SD)** | 14.1 (10.2)  1 missing | 15.3 (10.1)  21 missing | 0.48 |
| **Viral Onset N(%)** |  |  | 0.39 |
| **Yes** | 24 (48.0) | 71 (48.6) |  |
| **No** | 17 (34.0) | 34 (24.0) |  |
| **Unsure** | 8 (16.0) | 33 (21.9) |  |
| **No Data** | 1 (2.0) | 8 (5.5) |  |
| **Physical Functioning, mean (SD)** | 44.0 (21.9)  1 missing | 40.0 (21.5)  4 missing | 0.27 |
| **Cognitive Functioning, mean (SD)** | 56.8 (23.5)  1 missing | 54.6 (21.4)  4 missing | 0.54 |
| **Fukuda Symptoms N(%)** |  |  |  |
| **Impaired Memory or Concentration** |  |  | 0.06 |
| **Yes** | 49 (98.0) | 139 (95.2) |  |
| **No** | 0 (0) | 7 (4.8) |  |
| **Unknown** | 1 (2.0) | 0 (0.0) |  |
| **Sore Throat** |  |  | 0.15 |
| **Yes** | 27 (54.0) | 95 (65.1) |  |
| **No** | 23 (46.0) | 47 (32.2) |  |
| **Unknown** | 0 (0) | 4 (0.03) |  |
| **Tender Cervical or Axillary Lymph Nodes** |  |  | 0.06^d^ |
| **Yes** | 24 (48.0) | 98 (67.1) |  |
| **No** | 3 (6.0) | 44 (30.1) |  |
| **Unknown** | 23 (46.0) | 4 (2.7) |  |
| **Muscle Pain** |  |  | 0.87 |
| **Yes** | 47 (94.0) | 131 (89.7) |  |
| **No** | 3 (6.0) | 13 (8.9) |  |
| **Unknown** | 0 (0) | 2 (1.4) |  |
| **Multi-joint Pain** |  |  | 0.84 |
| **Yes** | 33 (66.0) | 102 (69.9) |  |
| **No** | 16 (32.0) | 40 (27.4) |  |
| **Unknown** | 1 (2.0) | 4 (2.7) |  |
| **New Headaches** |  |  | 0.82 |
| **Yes** | 38 (76.0) | 103 (70.5) |  |
| **No** | 11 (22.0) | 40 (27.4) |  |
| **Unknown** | 1 (2.0) | 3 (2.1) |  |
| **Unrefreshing Sleep** |  |  | 0.78 |
| **Yes** | 48 (96.0) | 141 (96.6) |  |
| **No** | 2 (4.0) | 3 (2.1) |  |
| **Unknown** | 0 (0) | 2 (1.4) |  |
| **Post-exertional Malaise** |  |  | 0.02 |
| **Yes** | 46 (92.0) | 145 (99.3) |  |
| **No** | 4 (8.0) | 1 (0.6) |  |
| **Unknown** | 0 (0) | 0 (0.7) |  |

SD = Standard Deviation, MFI-20 = Multidimensional Fatigue Inventory 20, FSS = Fatigue Severity Scale

^a^ p values are from t-tests for continuous variables or from the Freeman-Halton extension of Fisher’s exact test for categorical variables with low cell counts.

^b^ A total of 200 subjects were recruited for the Stanford University Genetic Expression and Immune System Dynamics study. Fifty subjects deferred participation in the survey while 150 participated. Out of this 150, 146 answered items related to post-exertional malaise. For each study recruitment question, the number “missing” (e.g. 1 subject out of 146 did not give their age) are the numbers of subjects who could not give an answer for that item.

^d^ p values are estimated using Monte Carlo methods due to low cell counts
